# Distribution of haemoglobin genotypes, knowledge, attitude and practices towards sickle cell disease among unmarried youths in the Buea Health District, Cameroon

**DOI:** 10.11604/pamj.2020.37.109.17864

**Published:** 2020-10-01

**Authors:** Nini Yesih Ngwengi, Peter Nde Fon, Dora Mbanya

**Affiliations:** 1 Department of Medicine, Faculty of Health Sciences, University of Buea, Buea, South West Region, Cameroon,; 2 Faculty of Health Sciences, University of Bamenda, Bamenda, Cameroun,; 3 Haematology & Blood Transfusion Service, Yaoundé University Teaching Hospital, Yaoundé, Cameroon

**Keywords:** Haemoglobin, knowledge, attitude, practice, sickle cell disease

## Abstract

**Introduction:**

sickle cell disease (SCD) is one of the commonest genetic causes of morbidity and mortality in the world. In resource-limited settings, SCD prevention through public education and screening could be a significant strategy to curb its prevalence. The study aimed at determining the distribution of haemoglobin genotypes among unmarried youths in Buea, Cameroon as well as their knowledge, attitude and practices towards SCD.

**Methods:**

a community-based, analytic, cross-sectional study was conducted within the city of Buea. Data was collected from 410 youths using self-administered questionnaires. Of the 410 youths, 100 were selected by purposive random sampling and their haemoglobin genotyping was done using haemoglobin electrophoresis. The data was analysed using the statistical software Epi Info Version 7.

**Results:**

the majority (51.5%) of the 410 respondents were females. The modal age range was 18- 21 years (46.8%) and 60.4% had tertiary education. Less than one quarter (20.5%) had good knowledge of SCD. Only 13.2% knew their genotype and 59.3% were willing to avoid carrier marriages. Out of the 100 participants for genotyping, 84.0% had normal haemoglobin (HbAA) and 16.0% had the sickle cell trait (HbAS).

**Conclusion:**

most of the respondents had moderate knowledge of SCD. Only a few knew their haemoglobin genotype and more than half were willing to avoid carrier marriages. The prevalence of sickle cell trait is high in Buea. The promotion of preventive methods like public education and genetic screening is recommended to reduce the burden of SCD in Cameroon.

## Introduction

Sickle cell disease (SCD) is a public health priority as declared by the World Health Organization (WHO) due its high birth prevalence [1]. Of the 332000 babies born with a major haemoglobinopathy worldwide every year, 275000 have SCD [2]. The disease is most frequently found in sub-Saharan Africa (SSA) where approximately 15 million people out of the estimated 25 million worldwide live [2, 3]. Sickle Cell Disease which has an autosomal recessive pattern of inheritance, consists of a group of disorders characterized by the presence of sickle haemoglobin (HbS). Haemoglobin (Hb) S is a structural variant of normal adult haemoglobin (HbA) caused by a mutation in the haemoglobin gene [3]. The term SCD refers to any condition in which greater than 50% of HbS is present leading to pathological consequences. The most common form (> 70%) of SCD worldwide results from the homozygous inheritance of the beta-globin subunit mutation and is usually referred to as either ‘SCD SS’ or as ‘sickle cell anaemia’ which is also the most severe form of the disease [4]. Over 700 structural haemoglobin variants exist but only two (HbS and HbC) are common in Africa [4, 5]. Sickle Cell Trait (SCT) or the carrier state is a heterozygous form characterized by the presence of up to 40% HbS, and the absence of anaemia [5].

When HbS is deoxygenated, the red blood cell (RBC) membrane is distorted producing the characteristic sickle-shaped cell [6]. These abnormal RBC´s then occlude small capillaries and venules therefore causing tissue ischemia, acute pain and gradual end organ damage [7]. As a chronic disease, the natural history of SCD is characterized by quiescent periods interspersed by acute events known as crisis, leading to patients seeking health care and frequent hospitalizations [3]. Africa has high mortality rates ranging from 50 to 90% for those aged less than 5 years [8]. This is due to a relative lack of several facilities including prenatal diagnostic services, systematic follow up and a broad range of life saving measures such as; routine penicillin prophylaxis, vaccination for common bacterial diseases and the provision of disease modifying treatment with Hydroxyurea. Poor access to hematopoietic stem cell transplantation (HSCT), which is the only potential curative therapy may also be contributory [3, 9-11].

Cameroon has a high carrier frequency of SCD (20-30%) as reported by WHO in 2006. It is the 6th country with the highest number of sickle cell births per year [1]. In spite of this, there is only one specialized centre for SCD in the capital city, Yaoundé. Prenuptial testing is available but few come forward for testing since health promotion to increase its awareness is still lacking. Also, care for people with SCD is suboptimal as majority of sufferers lack financial resources [12]. Although this aspect of control of the disease has not been given enough emphasis, prevention through public education, genetic counselling and screening is a better economic strategy especially in resource limited settings [13]. These preventive methods are reported to be effective in the control of thalassemia in Cyprus and Iran [14]. Nevertheless, appropriate knowledge regarding SCD is required for individuals, especially carriers to make informed decisions about their reproductive life [11]. Consequently studies are necessary to elucidate the epidemiology of SCD and the knowledge, attitude and practices towards SCD in Cameroon. Thus this study investigated the distribution of haemoglobin genotypes, knowledge, attitude and practices towards SCD among unmarried youths in Buea, Cameroon.

## Methods

The study was a community-based, analytic, cross-sectional study carried out from January to March, 2017 in Buea. Buea is the capital city of the South-West Region of Cameroon [15]. Education is one of the major activities the town is known for, with a large number of primary, secondary and tertiary educational institutions [16]. Most notable among the tertiary institutions is the University of Buea. Due to its position as a University town and the regional capital, there are a significant number of ethnic groups present. The Buea Health District is partitioned into seven health areas; Bova, Tole, Molyko, Bokwoango, Muea, Buea Road and Buea Town. All relevant administrative authorizations were obtained, as well as ethical clearance from the Institutional Review Board of the Faculty of Health Sciences of the University of Buea.

**Study population and sampling:** the study population was all unmarried youths between the ages of 18 and 35 years living in Buea during the study period. Purposive sampling was employed to obtain the desired number of participants from all seven health areas in the Buea Health District. Once in the various health areas, households were enrolled in accordance with a strict non-skip door to door survey protocol. A household was defined as a small group of persons who share the same living accommodation, who pool some or all of their income and wealth and who consume certain types of goods and share services collectively mainly housing and food. One eligible participant was then chosen from each household. If there was more than one eligible participant in a household, one was selected by simple random sampling (balloting). Based on sample size calculations in cross-sectional surveys [17], and using a proportion of 0.48 [18], a minimum sample size of 383.54 was estimated for the study. A total of 428 individuals were approached. However, 18 individuals refused to participate in the study. Out of the 410 respondents who completed the questionnaires, purposive random sampling was employed to select the desired number of individuals (100) for haemoglobin genotyping commensurately from all seven health areas. These individuals were reached via their mobile phones and directed to Solidarity Clinic, Buea where their blood samples were collected and haemoglobin genotyping by haemoglobin electrophoresis was performed.

**Knowledge, attitude and practice (KAP) study:** data was obtained by using a structured interviewer-administered questionnaire. The questionnaire contained data on socio demographic parameters and KAP related to the control of SCD. Each respondent´s age, gender, religion, level of education, and number of children were covered in the socio-demographic section. The section on knowledge included responses on aetiology, disease manifestations, control measures and management of SCD. This was composed of twelve questions. Structured closed ended questions offering choices of yes, no, and I don´t know were mostly used in the knowledge section. The attitudes and practices towards SCD referred to the degree of positive or negative agreements with statements concerning attitudes and beliefs towards the screening test, the impact of knowing the risk of having a child with the disease and the use of legislation to curb the prevalence of SCD. This section was composed of four questions. Respondents´ attitude and practices were assessed mostly on a three-point scale including responses such as agree, disagree and not decided. The questionnaire was pretested among twenty randomly selected youths in the community. The surveyors were trained in data collection before the study started. One team composed of one medical doctor and two medical students collected the household data. To avoid confusion, terminologies were explained to respondents during the data collection process.

**Haemoglobin genotyping:** one hundred subjects were recruited for haemoglobin genotyping. Two millilitres (2ml) of blood was collected from each participant into a labelled tri-potassium ethylene diamine tetra-acetic acid (k3EDTA) tube and mixed gently. Samples were processed within an hour after collection. The blood samples were centrifuged at 1500g for 5 minutes and the plasma removed. The red cells were then washed four times with normal saline (0.9%). Few drops of distilled water were added to lyse the red cells, after which four drops of carbon tetrachloride (CCL4) was added, vortexed and centrifuged at 1500g for 20 minutes to separate the haemoglobin. The hemolysates was transferred to a clean glass test tube. Each compartment of the cellulose acetate haemoglobin electrophoretic tank (Consort EV 243) was filled with Tris-EDTA Borate (TEB) buffer (pH 8.6) to a depth of about 2.5cm. The cellulose acetate membrane was impregnated with TEB buffer and blotted with tissue paper to remove excess buffer but not allowed to dry. By means of an applicator, the control and test hemolysates samples were applied on the cellulose acetate membrane and carefully introduced onto the frame of the electrophoretic tank, with both ends in contact with the buffer. The lid of the tank was replaced and the tank connected to a power supply of 250 volts and current 50mA and allowed to run for 30 minutes. The power was disconnected and the membrane removed and allowed to air-dry. The results were read against the control (hemolysates from a sickle cell trait sample).

**Data analysis:** in order to facilitate data analysis, 12 of the questions in the knowledge section were scored 1 for the correct response and 0 for the wrong response. A score of 0 - 4 was considered poor knowledge, 5-8 fair knowledge, while 9-12 was considered good knowledge. Data was inputted and analysed using EPI Info Version 7. Descriptive and inferential analysis were performed and presented as frequency counts and percentages in tables. The mean SCD knowledge scores were compared between groups using one way analysis of variance (ANOVA). Probability values of less than 0.05 were considered to be statistically significant.

## Results

**Socio-demographic characteristics of the study population:** of 410 youths participating, 211 (51.5%) were females and 199 (48.5) were males (Table 1). The overall mean age was 21 ± 3.4 years and the age group of 18 - 21 was most represented (46.8%). Most participants (84.6%) did not have children and the majority (99.5%) were Christians. Concerning their educational status, 4.9% had only primary education, 34.7% had secondary education and 60.4% had tertiary education.

**Table 1:** socio demographic characteristics of respondents

Variable (n = 410)	Frequency	Percentage
**Age**		
18-21	192	46.8
22-25	149	36.3
26-30	42	10.2
31-35	27	6.6
**Have children**		
Yes	63	15.4
No	347	84.6
**Gender**		
Male	199	48.5
Female	211	51.5
**Religion**		
Christianity	408	99.5
Islam	2	0.5
Others	0	0.0
**Level of education**		
Primary	20	4.9
Secondary	142	34.7
Tertiary	248	60.4

**Awareness of sickle cell disease:** most of the participants (89.8%) had heard about Sickle cell disease while the remaining 10.2% were not aware of the disorder. The main channel of information (41.7%) was through formal learning. Other sources of information included: affected persons (21.0%), media (20.2%) and health professionals (15.4%). About one half (53%) of respondents claimed to have relations or friends having SCD.

**Knowledge of sickle cell disease:** most of the respondents (44.9%) had moderate knowledge of SCD (Table 2). Interestingly 34.6% had poor knowledge of SCD. The mean knowledge score was 5.55 ± 3.08.

**Table 2:** level of knowledge of SCD

Variable (n = 410)	Frequency	Percentage
**Level of Knowledge**		
0-4 (Poor)	142	33.6
5-8 (Moderate)	184	44.9
9-12 (Good)	84	20.5

Mean knowledge score = **5.5512 ± 3.0758**

**Attitude and practices towards sickle cell disease:** the majority of participants (86.8%) did not know their haemoglobin genotype (SCD carrier status). Of the 48 (13.2%) respondents who claimed to know their haemoglobin genotype, the commonest reason for checking was personal curiosity (36.1%). The most reported haemoglobin variant among participants was HbAA (85.1%) while 14.9% were HbAS.

A few of the respondents (13.2%) expressed willingness to marry another individual with sickle cell trait despite the risk of having children with SCD. More than half (59.3%) were unwilling to take such risks while 27.5% were undecided. Only 8.2% of participants will opt for termination of pregnancy compared to 43.2% who will allow the pregnancy to continue if they discover their unborn child had SCD. The remaining 48.6% were undecided at the time of the study. More than half of participants (50.8%) disagreed with the use of legislation against marital union between people with sickle cell trait to prevent further births of SCD babies.

**Factors associated with having higher knowledge of SCD:** having a higher level of education and being exposed to formal learning about SCD were significantly associated with good knowledge of SCD (p value < 0.05). Being female, knowing someone with SCD and knowledge of one´s own haemoglobin genotype were all found to be associated with higher knowledge (p value < 0.05).

**Distribution of haemoglobin genotypes**: out of the 100 participants for which haemoglobin genotyping was done, 38 were male and 62 were female. Eighty-four (84) of 100 participants were AA and the remaining 16 were AS (Table 3). Of the 16 participants who were AS, 4 (25.0%) were male and 12 (75%) were female.

**Table 3:** distribution of haemoglobin genotypes

Variable (n = 100)	Frequency	Percentage
HbAA	84	84.0
HbAS	16	16.0
HbSS	0	0.0

## Discussion

Quantitative studies provide essential evidence on which public health decisions can be made [19]. This study showed that about 90% of our study participants had heard about SCD suggesting a good level of awareness. This finding is similar to that of studies done in urban areas in Nigeria, Ghana and USA [11, 20-23] but less than the 46.2% obtained in a study conducted in a rural area in India [24]. The high level of awareness in the studies done in the urban areas may be due to the fact that the respondents are likely to have access to opportunities like mass media and internet which could broaden their knowledge about diseases unlike in rural areas where the youths have limited access to mass media and internet. The commonest medium through which participants got information on SCD in this study was through formal learning. This is in contrast to studies conducted in Ghana and Nigeria [11, 21] where the main source of information of respondents was from the media.

However, in our study, knowledge of SCD among youths was found to be moderate. These results concurred with that of previous studies conducted in USA, Nigeria and Ghana [11, 21, 25]. However, these results were contrary to those of other studies which either showed poor knowledge of SCD among youths [5, 13, 18, 26, 27] or good knowledge of SCD among youths [19, 22, 23]. Participants who had a higher level of education (secondary and tertiary education) and who had been exposed to formal learning on SCD had significantly higher knowledge scores (p < 0.05). This association compares with other studies done in Africa [11, 19, 27, 28]. This is an expected association because SCD is probably taught in some courses in a number of secondary schools, high schools and universities across Africa. About half (52.99%) of the participants knew someone having SCD implying that the disease is not uncommon. In this study, when compared, knowing one´s haemoglobin genotype and having a high knowledge score was significantly associated (p < 0.05). This could be explained by the fact that accurate knowledge on SCD may have been given to them by the healthcare providers who requested for or conducted the test.

Concerning attitudes and practices toward SCD, in our study, only 13.2% of respondents claimed that they knew their haemoglobin genotype. This percentage (13.2%) is much lower compared to the 94.6% among 370 postgraduates in Nigeria [11] and the 25.0% among 320 youth workers in Ghana [19]. Many other studies had much higher percentages [13, 18, 20, 25, 27]. On the other hand, our study found a higher percentage compared to the 2.7% among 150 people in Ghana [29]. In our study, curiosity was observed to be the commonest indication for carrier status check. In a similar study in Nigeria [11], school entry and doctor´s request were the commonest indicators. More than half (59.34%) of respondents were averse to union of trait carriers. This percentage is slightly higher than the 50% obtained among youth workers in Nigeria [27] but much lower than the 78% obtained among public servants in Ghana [21]. This difference in results could be explained by different levels of knowledge on SCD among the various respondents since knowledge on SCD could greatly influence one´s attitude towards it as shown in a study in Sudan by Daak *et al*. [19].

Less than half (43.7%) of respondents were averse to the termination of an affected pregnancy while 8.2% will agree to selective abortion if permitted by law. Up to 38.1% in Nigeria [11] will agree to selective abortions. Less than half (28.5%) of respondents agreed that legislation against carrier marriages might be engaged in SCD control in Cameroon. This finding is lower than the 54.6% in Nigeria [11] who agreed to the use of legislation against carrier marriages. However, a study conducted in Greece showed that the use of punitive measures in the control of thalassemia gave disappointing results, as it led to increased anxiety, stigmatization, denial and falsification of results [14]. The distribution of haemoglobin genotype in our study was (84.0% with HbAA and 16.0% with HbAS) different from that in another study in Cameroon which showed a higher percentage of HbAS (26.4%) and the presence of HbSS (2.9%) among 929 participants among all age groups [30].The complete absence of HbSS in our study is probably due to fact that our study was conducted among youths between the ages 18 and 35 and by this age, most cases of SCD must have already been diagnosed. Also, the small sample size (100 participants) could further explain this. The small sample size could also account for the lower prevalence of HbAS found in our study.

## Conclusion

This study illustrates that knowledge, attitude and practices towards SCD among youths in Buea is insufficient as only few respondents had good knowledge of SCD. It also showed a relatively high prevalence of Sickle Cell Trait in the study population. With the presence of very few specialized centres with multi-specialist teams dedicated to the care of persons with SCD in Cameroon, primary prevention through health education, population screening, genetic counselling and avoidance of carrier marriages remain the most cost effective methods of curbing the disease in sub-Saharan Africa, and Cameroon in particular.

### What is known about this topic

- This study provides information on KAP regarding SCD among youths prior to marriage, in Buea, Cameroon;
- We also studied factors influencing knowledge on SCD;
- This study also shows the prevalence of sickle cell trait specifically among youths in Buea, Cameroon.

### What this study adds

- This study provides information on KAP regarding SCD among youths prior to marriage, in Buea, Cameroon;
- We also studied factors influencing knowledge on SCD;
- This study also shows the prevalence of sickle cell trait specifically among youths in Buea, Cameroon.
